# A real-world pharmacovigilance study of FDA adverse event reporting system events for Capmatinib

**DOI:** 10.1038/s41598-024-62356-w

**Published:** 2024-05-18

**Authors:** Yiming Qi, Jing Li, Sisi Lin, Shuangshuang Wu, Kequn Chai, Xin Jiang, Jiancheng Qian, Cheng Jiang

**Affiliations:** 1 Tongde Hospital Affiliated to Zhejiang Chinese Medical University (Tongde Hospital of Zhejiang Province), No. 234, Gucui Road, Hangzhou, 310012 Zhejiang China; 2 Zhejiang Academy of Traditional Chinese Medicine, Tongde Hospital of Zhejiang Province, Hangzhou, 310012 Zhejiang China; 3 Department of Traditional Chinese Medicine, The First Affiliated Hospital of Wenzhou Medical University, Wenzhou, 325000 Zhejiang China; 4 Wenling Hospital of Traditional Chinese Medicine Affiliated to Zhejiang, Chinese Medical University, Wenling, 317500 Zhejiang China

**Keywords:** Targeted therapies, Non-small-cell lung cancer, Drug safety

## Abstract

Capmatinib is a potent selective mesenchymal-epithelial transition inhibitor approved in 2020 for the treatment of metastatic non-small cell lung cancer. As real-world evidence is very limited, this study evaluated capmatinib-induced adverse events through data mining of the FDA Adverse Event Reporting System database. Four disproportionality analysis methods were employed to quantify the signals of capmatinib-related adverse events. The difference in capmatinib-associated adverse event signals was further investigated with respect to sex, age, weight, dose, onset time, continent, and concomitant drug. A total of 1518 reports and 4278 adverse events induced by capmatinib were identified. New significant adverse event signals emerged, such as dysphagia, dehydration, deafness, vocal cord paralysis, muscle disorder, and oesophageal stenosis. Notably, higher risk of alanine aminotransferase and aspartate aminotransferase increases were observed in females, especially when capmatinib was combined with immune checkpoint inhibitors. Compared with Europeans and Asians, Americans were more likely to experience peripheral swelling, especially in people > 65 years of age. Renal impairment and increased blood creatinine were more likely to occur with single doses above 400 mg and in Asians. This study improves the understanding of safety profile of capmatinib.

## Introduction

Lung cancer poses a leading and formidable oncological challenge as the primary cause of cancer mortality worldwide^1^. Non-small cell lung cancer (NSCLC) accounts for approximately 80–85% of all lung cancer cases, with its aggressive nature posing substantial challenges to effective management^2^. The mesenchymal-epithelial transition (MET) gene, encoding the MET receptor tyrosine kinase, plays a crucial role in regulating cell growth, survival, and motility. Patients harboring MET exon 14 skipping mutations exhibit unique clinical characteristics, often presenting with advanced-stage NSCLC. The prevalence of these mutations among NSCLC patients ranges from 3 to 4%^3,4^. Studies have revealed that patients with NSCLC and MET exon 14 skipping mutations face distinct prognostic challenges, often experiencing shorter overall survival and increased tumor progression risk^4^.

Historically, traditional chemotherapy has been the primary treatment approach for NSCLC patients with MET exon 14 skipping mutations. However, the efficacy of chemotherapy in this context has proven limited. In response, targeted and immunotherapy approaches have entered to address the specific vulnerabilities associated with MET exon 14 skipping mutations. These precision approaches have revolutionized the landscape of lung cancer treatment, especially in situations where traditional treatments may be less effective. Nevertheless, the overall effectiveness of these therapies still remains unsatisfactory^5^. These challenges highlight the importance of ongoing drug development and post-marketing monitoring to continually improve treatment outcomes.

Capmatinib, a highly selective MET inhibitor, gained approval in 2020 for treating MET-mutated NSCLC^6,7^. The National Comprehensive Cancer Network NSCLC guidelines recommend capmatinib as either a first-line therapy or subsequent therapy option (preferred) for patients with metastatic NSCLC and MET exon 14 skipping mutations based on clinical trial data and FDA approval^8^. Capmatinib may be used as a subsequent therapy option if it, tepotinib, or crizotinib were not previously given as first-line therapy^8^. A non-randomized, open-label, multicenter phase II trial GEOMETRY mono1 clinical trials demonstrated significant antitumor activity of capmatinib in this population^9^. The overall response rate was 44% for previously treated patients and 68% for untreated patients, with median durations of response at 9.7 months and 16.6 months, respectively^10^. These findings highlight substantial antitumor activity of capmatinib in advanced NSCLC patients harboring MET exon 14 skipping mutations.

Despite encouraging efficacy, capmatinib is associated with adverse events like any therapy. Current understanding of capmatinib adverse events primarily stems from clinical trials. Nevertheless, clinical trials may not fully capture real-world reactions due to strict designs, limited samples and follow-up, and controlled conditions that differ from clinical practice after drug launch. Consequently, the capmatinib adverse event profile remains inadequately defined. Furthermore, a clinical study of 364 capmatinib-treated patients reported 48 serious adverse events leading to 39 discontinuations^10^. One death from capmatinib-linked pneumonia was also reported^10^. Comprehensive anticipation and timely management of capmatinib-associated adverse events are therefore essential to minimize potential risks.

The FDA Adverse Event Reporting System (FAERS) is one of the world's largest pharmacovigilance databases, comprising voluntary reports on FDA-approved therapies^11^. In this study, the clinical safety of capmatinib was investigated based on the FAERS database. The clinical characteristics of the capmatinib-associated adverse events were analyzed. The potential adverse event signals of capmatinib were explored. Furthermore, the difference in capmatinib-associated adverse event signals was investigated concerning sex, age, weight, dose, onset time, continent, and concomitant drug. This study provides comprehensive evaluation for the safety of capmatinib in real-world clinical use.

## Results

### Clinical characteristics analysis

**Figure 1:**
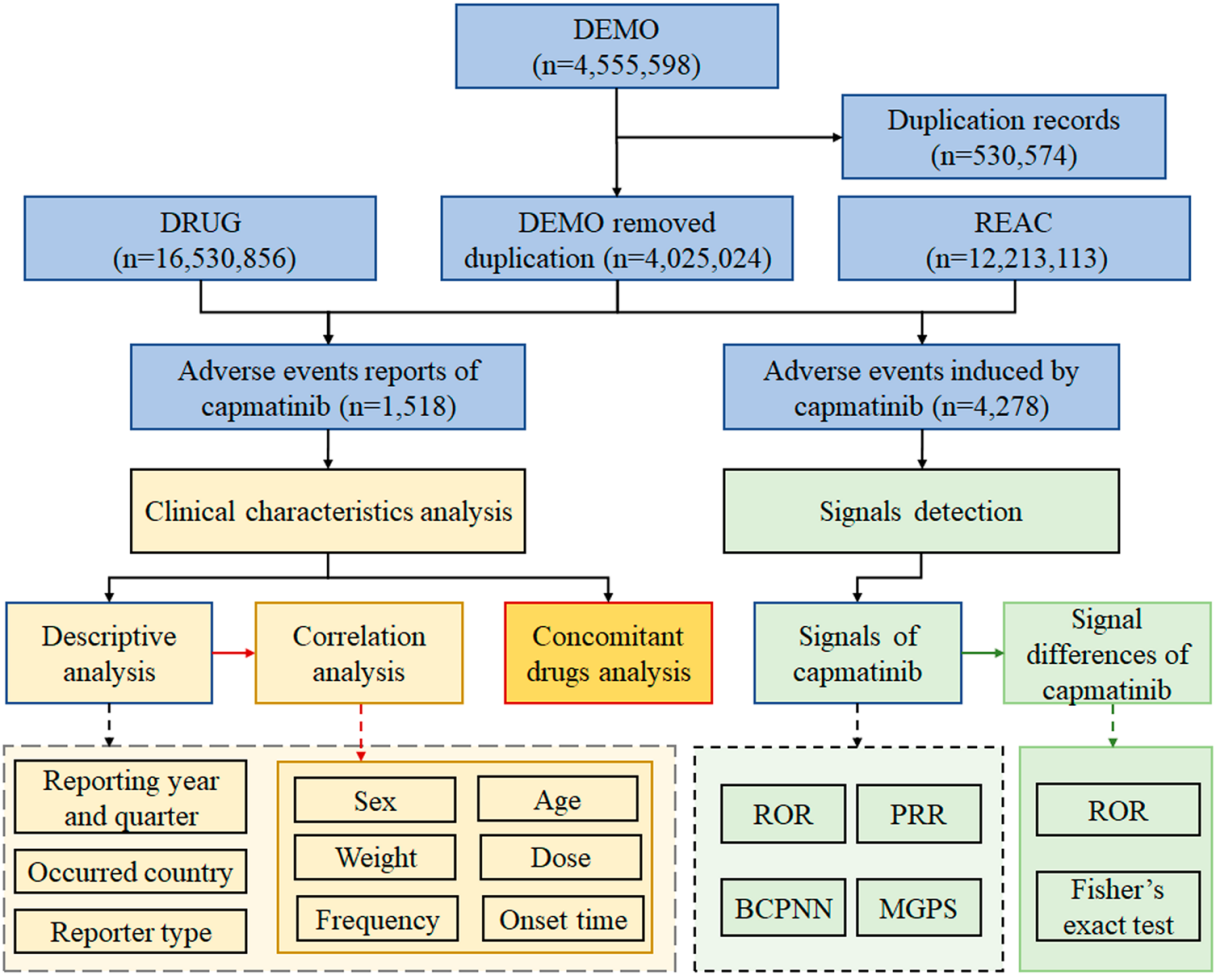
Flow diagram of data collection and analysis of capmatinib-associated adverse events.

A total of 4,555,598 adverse event reports were obtained from the DEMO dataset initially. Duplicate reports were identified and removed, eliminating 530,574 cases. Statistical analysis was then performed on the remaining 4,025,024 adverse event reports after duplicate removal. After matching the DRUG dataset with the DEMO and REAC datasets, 1518 reports and 4278 adverse events with capmatinib as the primary suspected (PS) drug were identified. The data collection and analysis workflow for capmatinib-associated adverse events is shown in Fig. 1.

**Figure 2:**
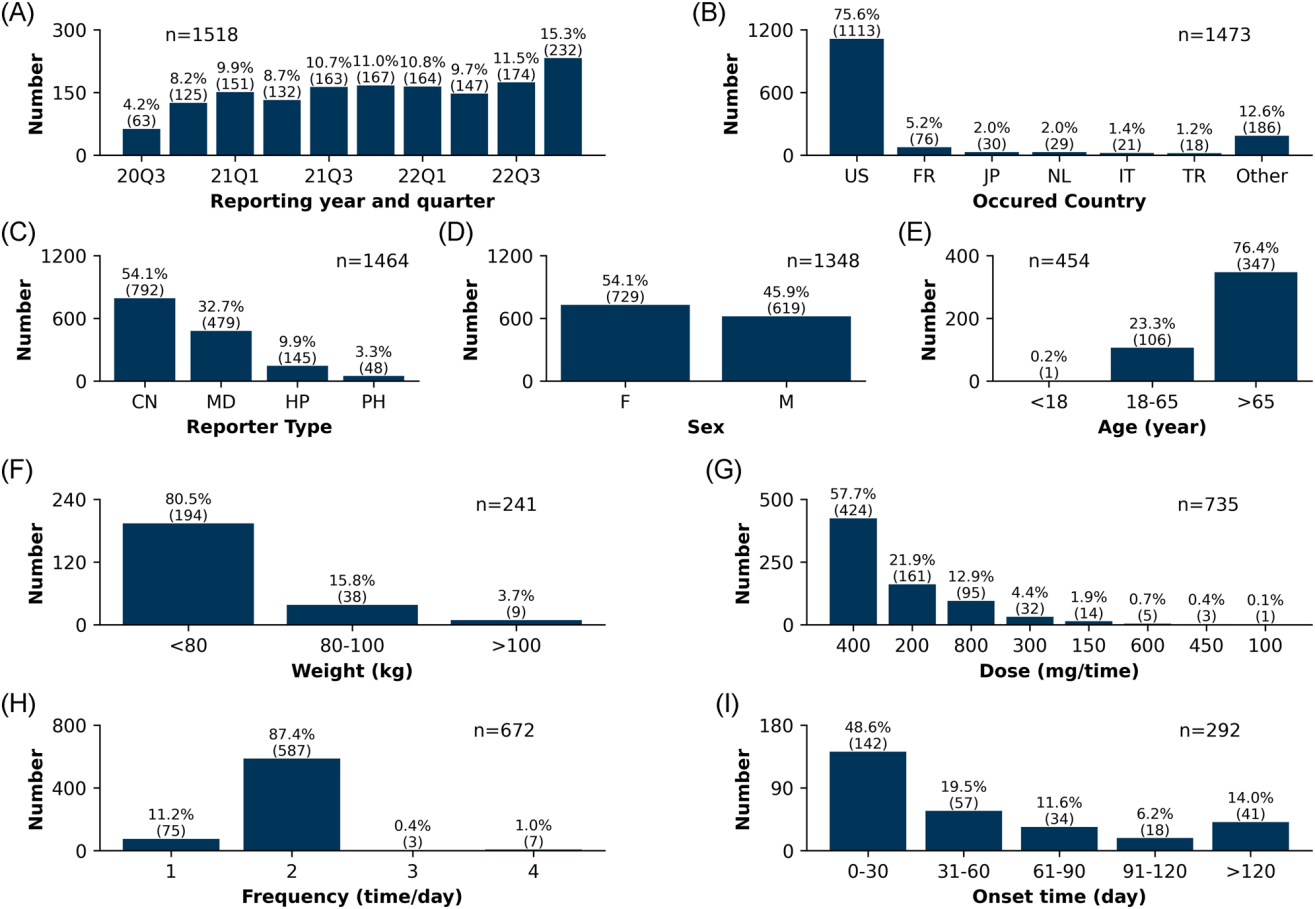
Clinical characteristics of capmatinib-associated adverse events. (**A**) Reporting year and quarter. (**B**) Occurred country. (**C**) Reporter type. (**D**) Sex. (**E**) Age. (**F**) Weight. (**G**) Dose. (**H**) Frequency. (**I**) Onset time.

The clinical characteristics of the 1518 capmatinib-associated adverse event reports are shown in Fig. 2. Overall, the number of capmatinib-associated adverse event reoprts gradually increased from the third quarter of 2020 to the fourth quarter of 2022. Regarding the countries where the events occurred, the United States reported 75.6% (n = 1113) of the adverse event reoprts, followed by 5.2% (n = 76) in France, and 2.0% (n = 30) in Japan. Excluding 54 reports with unknown reporters, consumers reported the most adverse event reports at 54.1% (n = 792). Sex data were available for 1348 cases. Among these, females accounted for 54.1% (n = 729) and males accounted for 45.9% (n = 619). Age data were reported in 454 cases, ranging from 16 to 86 years. The majority of patients with reported ages were > 65 years old (76.4%, n = 347). Effective weight data (wt_cod as KG) were available for 241 patients. The majority of patients weighed < 80 kg (80.5%, n = 194). Excluding missing data and incomparable doses, 735 effective dose cases (dose_unit as MG) were available. It was found that 400 mg (57.7%, n = 424) accounted for the vast majority of doses, followed by 200 mg (21.9%, n = 161) and 800 mg (12.9%, n = 95). In terms of frequency, 672 effective cases were available. Among these, 87.4% (n = 587) were taken at the correct frequency of twice daily (including BID and Q12H), followed by once daily (including QD and HS) 11.2% (n = 75). Excluding erroneous reports, inaccurate date entry and missing data, a total of 292 reports described the valid onset time of capmatinib-associated adverse events. Among these, 48.6% (n = 142) of adverse events occurred within the first month of administration, followed by 19.5% (n = 57) occurring in the second month. 14.0% (n = 41) of cases still occurred after 4 months of administration.

**Figure 3:**
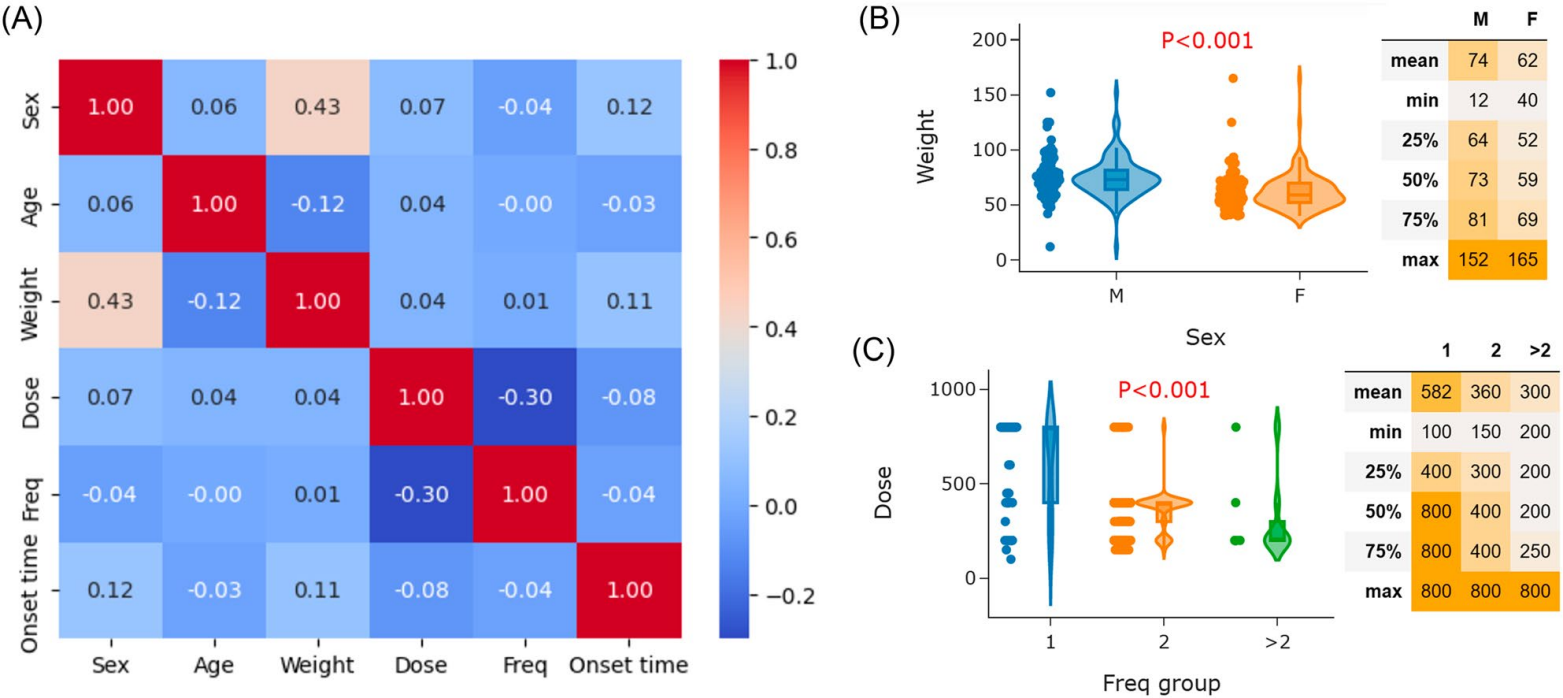
Correlation between typical clinical characteristics of capmatinib-associated adverse events. (**A**) Spearman correlation coefficients between sex, age, weight, dose, frequency, and onset time. (**B**) Violin plot and the T-test result of weight between males and females. (**C**) Violin plot and the ANOVA test result of dose between frequency groups.

The Spearman correlation coefficients for typical clinical characteristics are presented in Fig. 3A. As depicted in Fig. 3A, there are strong positive correlations observed for sex/weight and negative correlations for dose/frequency. Figure 3B displays the violin plots and T-test results comparing weight between males and females. A statistically significant difference in weight (74 vs. 62 kg; *P* < 0.001) was found between males and females. This result was related to the weight characteristics of the population using capmatinib. Furthermore, the analysis of dose across frequency groups is illustrated in Fig. 3C, presenting the violin plot and Analysis of Variance (ANOVA) result. Significant differences in dose were observed among the three frequency groups (582 vs. 360 vs. 300 mg; *P* < 0.001) for once daily, twice daily, and three to four times daily regimens. It was noteworthy that patients taking high doses of capmatinib (> 400 mg) mainly followed a once-daily frequency, which is not clinically recommended. These findings suggest close attention should be paid to the rational use of capmatinib, including appropriate dose and frequency.

**Figure 4:**
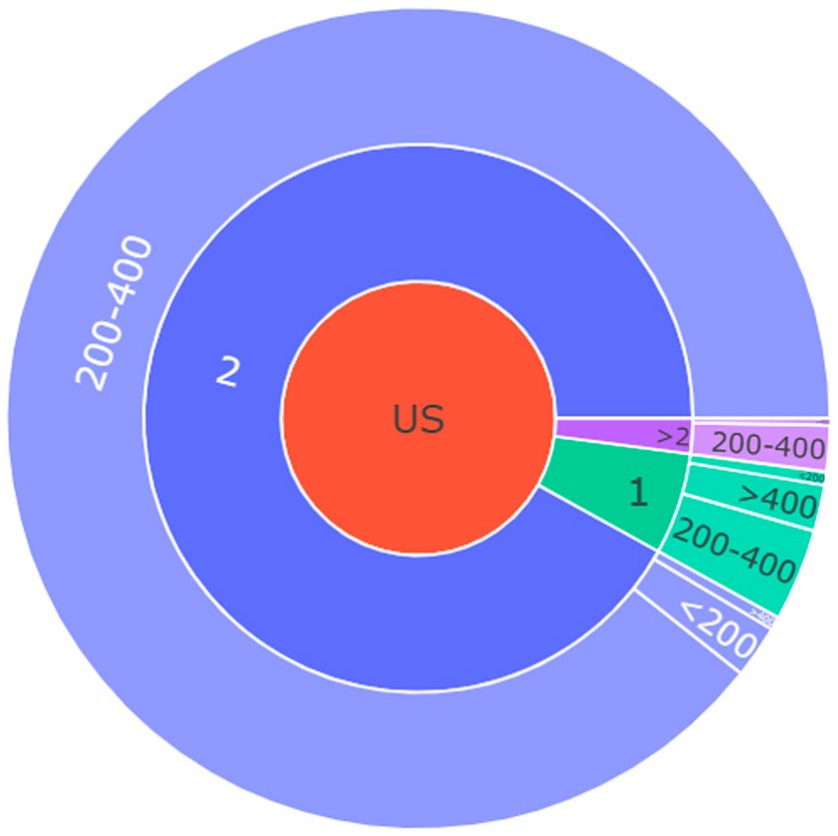
Sunburst plot of cases number by dose and frequency in the United States.

The United States reported the most adverse event reports that concurrently recorded dose and frequency (n = 385). The sunburst plot of case numbers by dose and frequency for the United States is shown in Fig. 4. Although 89.4% (n = 344) of cases in United States used the proper 200–400 mg twice daily dose and frequency, over 10% still had incorrect administration, such as 3.6% (n = 14) taking 200–400 mg once daily and 1.8% (n = 7) taking > 400 mg once daily. France had the second highest number of reported cases, but only 44 cases concurrently reported dose and frequency.

**Figure 5:**
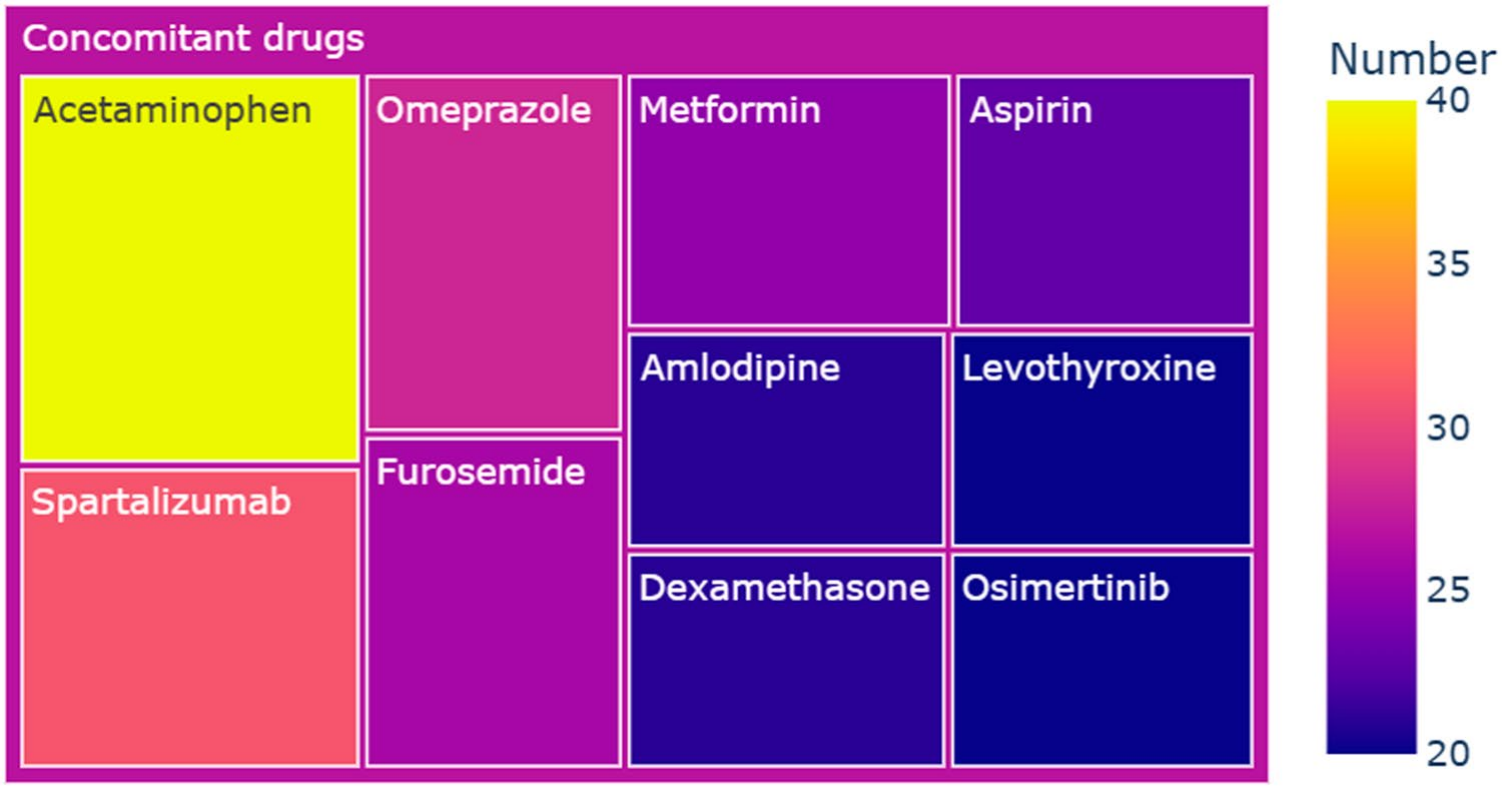
Top 10 concomitant drug occurrences within the capmatinib-associated adverse event reports.

The concomitant drugs recorded in adverse event reports linked to capmatinib were highly diverse, encompassing 454 distinct medications. Figure 5 delineates the top 10 concomitant drug occurrences within the capmatinib-associated adverse event reports. Acetaminophen, spartalizumab, and omeprazole were the most frequently concomitant drugs, accounting for 2.6% (n = 40), 2.0% (n = 31) and 1.8% (n = 28), respectively. In addition to the spartalizumab, capmatinib was also combined with other immune checkpoint inhibitors, including pembrolizumab at 1.2% (n = 18), atezolizumab at 0.1% (n = 1), and durvalumab at 0.1% (n = 1).

### Signals detection

The case number and signal strength of capmatinib-related adverse events at the System Organ Class (SOC) level are described in Table 1. Statistically, it was found that capmatinib-associated adverse events involved 26 SOCs. A total of 8 SOCs met the criteria of at least one of the four algorithms, including general disorders and administration site conditions (SOC: 10,018,065), gastrointestinal disorders (SOC: 10,017,947), neoplasms benign, malignant and unspecified (incl cysts and polyps) (SOC: 10,029,104), respiratory, thoracic and mediastinal disorders (SOC: 10,038,738), investigations (SOC: 10,022,891), metabolism and nutrition disorders (SOC: 10,027,433), hepatobiliary disorders (SOC: 10,019,805), and ear and labyrinth disorders (SOC: 10,013,993).

**Table 1:** Case number and signal strength of capmatinib-related adverse events at the SOC level.

SOC	a	b	c	d	ROR		PRR		BCPNN		MGPS	
					ROR	Lower limit of 95% CI	PRR	χ2	IC	IC025	EBGM	EBGM05
General disorders and administration site conditions (SOC: 10,018,065) ^13^	1379	2899	2,142,077	10,066,758	2.24	2.10	1.84	637.70	0.88	0.79	1.84	1.72
Gastrointestinal disorders (SOC: 10,017,947) ^13^	482	3796	938,209	11,270,626	1.53	1.39	1.47	77.35	0.55	0.41	1.47	1.33
Neoplasms benign, malignant and unspecified (incl cysts and polyps) (SOC: 10,029,104)^13^	322	3956	669,224	11,539,611	1.40	1.25	1.37	34.53	0.46	0.29	1.37	1.23
Respiratory, thoracic and mediastinal disorders (SOC: 10,038,738)^13^	319	3959	540,141	11,668,694	1.74	1.55	1.69	92.99	0.75	0.58	1.69	1.50
Investigations (SOC: 10,022,891)^13^	290	3988	729,096	11,479,739	1.14	1.02	1.14	4.96	0.18	0.01	1.14	1.01
Nervous system disorders (SOC: 10,029,205)	194	4084	868,556	11,340,279	0.62	0.54	0.64	43.06	− 0.65	− 0.86	0.64	0.55
Injury, poisoning and procedural complications (SOC: 10,022,117)	188	4090	1,452,911	10,755,924	0.34	0.29	0.37	229.85	− 1.44	− 1.65	0.37	0.32
Metabolism and nutrition disorders (SOC: 10,027,433) ^123^	164	4114	224,588	11,984,247	2.13	1.82	2.08	94.13	1.06	0.82	2.08	1.78
Musculoskeletal and connective tissue disorders (SOC: 10,028,395)	161	4117	640,039	11,568,796	0.71	0.60	0.72	18.83	− 0.48	− 0.71	0.72	0.61
Skin and subcutaneous tissue disorders (SOC: 10,040,785)	128	4150	596,889	11,611,946	0.60	0.50	0.61	33.10	− 0.71	− 0.96	0.61	0.51
Infections and infestations (SOC: 10,021,881)	101	4177	667,754	11,541,081	0.42	0.34	0.43	79.94	− 1.21	− 1.49	0.43	0.35
Renal and urinary disorders (SOC: 10,038,359)	86	4192	238,468	11,970,367	1.03	0.83	1.03	0.07	0.04	− 0.27	1.03	0.83
Hepatobiliary disorders (SOC: 10,019,805)^123^	82	4196	94,610	12,114,225	2.50	2.01	2.47	72.48	1.31	0.96	2.47	1.99
Psychiatric disorders (SOC: 10,037,175)	76	4202	707,295	11,501,540	0.29	0.23	0.31	126.45	− 1.7	− 2.02	0.31	0.24
Vascular disorders (SOC: 10,047,065)	67	4211	225,364	11,983,471	0.85	0.66	0.85	1.85	− 0.24	− 0.59	0.85	0.67
Cardiac disorders (SOC: 10,007,541)	51	4227	234,907	11,973,928	0.62	0.47	0.62	12.14	− 0.69	− 1.08	0.62	0.47
Ear and labyrinth disorders (SOC: 10,013,993) ^123^	38	4240	48,229	12,160,606	2.26	1.64	2.25	26.43	1.17	0.66	2.25	1.63
Blood and lymphatic system disorders (SOC: 10,005,329)	34	4244	204,894	12,003,941	0.47	0.33	0.47	20.23	− 1.08	− 1.55	0.47	0.34
Eye disorders (SOC: 10,015,919)	30	4248	226,784	11,982,051	0.37	0.26	0.38	31.37	− 1.41	− 1.90	0.38	0.26
Surgical and medical procedures (SOC: 10,042,613)	26	4252	166,870	12,041,965	0.44	0.30	0.44	18.28	− 1.17	− 1.70	0.44	0.30
Product issues (SOC: 10,077,536)	26	4252	216,126	11,992,709	0.34	0.23	0.34	33.24	− 1.54	− 2.06	0.34	0.23
Reproductive system and breast disorders (SOC: 10,038,604)	14	4264	69,246	12,139,589	0.58	0.34	0.58	4.37	− 0.79	− 1.50	0.58	0.34
Immune system disorders (SOC: 10,021,428)	10	4268	131,492	12,077,343	0.22	0.12	0.22	28.55	− 2.20	− 2.97	0.22	0.12
Endocrine disorders (SOC: 10,014,698)	4	4274	31,027	12,177,808	0.37	0.14	0.37	4.35	− 1.44	− 2.54	0.37	0.14
Congenital, familial and genetic disorders (SOC: 10,010,331)	4	4274	32,310	12,176,525	0.35	0.13	0.35	4.75	− 1.50	− 2.59	0.35	0.13
Social circumstances (SOC: 10,041,244)	2	4276	60,174	12,148,661	0.09	0.02	0.09	17.36	− 3.40	− 4.55	0.09	0.02

^1^SOCs met the criteria of ROR algorithm; ^2^SOCs met the criteria of PRR algorithm; ^3^SOCs met the criteria of BCPNN algorithm; ^4^SOCs met the criteria of MGPS algorithm.

A total of 65 signals at the Preferred Terms (PTs) level were detected after meeting the criteria of reporting odds ratio (ROR), proportional reporting ratio (PRR), Bayesian confidence propagation neural network (BCPNN), and multi-item gamma Poisson shrinker (MGPS) algorithms, simultaneously. 16 capmatinib-unrelated signals, including 12 signals of neoplasms benign, malignant and unspecified (incl cysts and polyps) (SOC: 10,029,104), 2 signals of product issues (SOC: 10,077,536), 1 signal of injury, poisoning and procedural complications (SOC: 10,022,117), and 1 signal of disease progression (PT: 10,061,818), were detected. The case numbers and signal strength of the capmatinib-unrelated adverse events at the PT level are listed in Supplementary Table S1.

After excluding the 16 capmatinib-unrelated adverse events, 49 significant disproportionality capmatinib-related adverse events remained, as shown in Table 2. In this study, signals such as peripheral swelling (PT: 10,048,959), fatigue (PT: 10,016,256), oedema peripheral (PT: 10,030,124), asthenia (PT: 10,003,549), nausea (PT: 10,028,813), and blood creatinine increased (PT: 10,005,483) were consistent with previous findings^12^. Additionally, death (PT: 10,011,906), dyspnoea (PT: 10,013,968), pleural effusion (PT: 10,035,598), blood albumin decreased (PT: 10,005,287), haemoptysis (PT: 10,018,964), and central nervous system lesion (PT: 10,051,290), might cause by the disease progression. Interestingly, some new significant signals were uncovered in the label and clinical trials, such as dysphagia (PT: 10,013,950), dehydration (PT: 10,012,174), deafness (PT: 10,011,878), vocal cord paralysis (PT: 10,047,674), muscle disorder (PT: 10,028,300), and oesophageal stenosis (PT: 10,030,194).

**Table 2:** Case number and signal strength of capmatinib-related adverse events at the PT level.

PT	a	b	c	d	ROR		PRR		BCPNN		MGPS	
					ROR	Lower limit of 95% CI	PRR	χ^2^	IC	IC025	EBGM	EBGM05
General disorders and administration site conditions (SOC: 10,018,065)												
Death (PT: 10,011,906)	268	4010	168,399	12,040,436	4.78	4.22	4.54	749.38	2.18	1.98	4.54	4.01
Peripheral swelling (PT: 10,048,959)	214	4064	38,739	12,170,096	16.54	14.41	15.77	2952.48	3.97	3.67	15.68	13.66
Fatigue (PT: 10,016,256)	186	4092	157,063	12,051,772	3.49	3.01	3.38	315.34	1.76	1.52	3.38	2.92
Oedema peripheral (PT: 10,030,124)	169	4109	15,005	12,193,830	33.42	28.63	32.14	5048.88	4.99	4.52	31.80	27.24
Oedema (PT: 10,030,095)	84	4194	7794	12,201,041	31.35	25.23	30.76	2394.13	4.93	4.18	30.44	24.50
Asthenia (PT: 10,003,549)	74	4204	65,283	12,143,552	3.27	2.60	3.23	114.74	1.69	1.31	3.23	2.57
Swelling (PT: 10,042,674)	36	4242	21,201	12,187,634	4.88	3.51	4.85	109.89	2.27	1.66	4.84	3.49
Generalised oedema (PT: 10,018,092)	20	4258	1731	12,207,104	33.12	21.29	32.97	613.08	5.03	3.07	32.61	20.96
Energy increased (PT: 10,048,779) *	4	4274	994	12,207,841	11.49	4.30	11.48	38.14	3.52	0.59	11.44	4.28
Concomitant disease aggravated (PT: 10,010,253)	4	4274	1274	12,207,561	8.97	3.36	8.96	28.20	3.16	0.49	8.94	3.35
Gastrointestinal disorders (SOC: 10,017,947)												
Nausea (PT: 10,028,813)	170	4108	134,151	12,074,684	3.72	3.19	3.62	324.98	1.85	1.61	3.61	3.10
Dysphagia (PT: 10,013,950) *	46	4232	14,693	12,194,142	9.02	6.74	8.93	323.52	3.16	2.51	8.91	6.66
Oesophageal stenosis (PT: 10,030,194) *	3	4275	510	12,208,325	16.80	5.40	16.79	44.28	4.06	0.31	16.70	5.36
Respiratory, thoracic and mediastinal disorders (SOC: 10,038,738)												
Dyspnoea (PT: 10,013,968)	91	4187	101,343	12,107,492	2.60	2.11	2.56	87.36	1.36	1.03	2.56	2.08
Pleural effusion (PT: 10,035,598)	25	4253	9575	12,199,260	7.49	5.05	7.45	139.38	2.89	2.01	7.43	5.02
Pneumonitis (PT: 10,035,742)	16	4262	5403	12,203,432	8.48	5.19	8.45	104.85	3.08	1.85	8.43	5.16
Pulmonary oedema (PT: 10,037,423)	14	4264	7421	12,201,414	5.40	3.19	5.38	49.91	2.43	1.31	5.38	3.18
Haemoptysis (PT: 10,018,964)	7	4271	4408	12,204,427	4.54	2.16	4.53	19.24	2.18	0.63	4.53	2.16
Pleurisy (PT: 10,035,618)	3	4275	777	12,208,058	11.03	3.55	11.02	27.23	3.46	0.20	10.98	3.53
Metabolism and nutrition disorders (SOC: 10,027,433)												
Decreased appetite (PT: 10,061,428)	71	4207	43,745	12,165,090	4.69	3.71	4.63	202.60	2.21	1.80	4.63	3.66
Fluid retention (PT: 10,016,807)	25	4253	8690	12,200,145	8.25	5.57	8.21	157.96	3.03	2.11	8.19	5.52
Dehydration (PT: 10,012,174) *	24	4254	20,068	12,188,767	3.43	2.29	3.41	40.96	1.77	1.06	3.41	2.28
Increased appetite (PT: 10,021,654)	8	4270	2506	12,206,329	9.13	4.56	9.11	57.59	3.18	1.29	9.08	4.54
Hypoalbuminaemia (PT: 10,020,942)	6	4272	1272	12,207,563	13.48	6.04	13.46	68.90	3.74	1.18	13.40	6.01
Musculoskeletal and connective tissue disorders (SOC: 10,028,395)												
Joint swelling (PT: 10,023,232)	68	4210	29,270	12,179,565	6.72	5.29	6.63	325.13	2.73	2.26	6.62	5.21
Muscle disorder (PT: 10,028,300) *	3	4275	1124	12,207,711	7.62	2.45	7.62	17.20	2.93	0.07	7.60	2.45
Investigations (SOC: 10,022,891)												
Blood creatinine increased (PT: 10,005,483)	39	4239	11,589	12,197,246	9.68	7.06	9.60	299.89	3.26	2.52	9.58	6.98
Alanine aminotransferase increased (PT: 10,001,551)	18	4260	9575	12,199,260	5.38	3.39	5.36	63.85	2.42	1.46	5.36	3.37
Aspartate aminotransferase increased (PT: 10,003,481)	16	4262	7769	12,201,066	5.90	3.61	5.88	64.67	2.55	1.49	5.87	3.59
Liver function test increased (PT: 10,077,692)	10	4268	5142	12,203,693	5.56	2.99	5.55	37.25	2.47	1.10	5.54	2.98
Blood bilirubin increased (PT: 10,005,364)	8	4270	3653	12,205,182	6.26	3.13	6.25	35.21	2.64	1.01	6.24	3.12
Blood sodium decreased (PT: 10,005,802) *	8	4270	3214	12,205,621	7.12	3.55	7.10	41.86	2.83	1.12	7.09	3.54
Creatinine renal clearance decreased (PT: 10,011,372)	8	4270	867	12,207,968	26.38	13.14	26.33	193.20	4.71	1.82	26.10	13.00
Blood albumin decreased (PT: 10,005,287)	6	4272	958	12,207,877	17.90	8.02	17.87	94.99	4.15	1.29	17.77	7.96
Gamma-glutamyltransferase increased (PT: 10,017,693)	6	4272	2865	12,205,970	5.98	2.68	5.98	24.82	2.58	0.71	5.97	2.68
Amylase increased (PT: 10,002,016) *	3	4275	644	12,208,191	13.30	4.28	13.29	33.95	3.73	0.26	13.24	4.26
Renal and urinary disorders (SOC: 10,038,359)												
Renal impairment (PT: 10,062,237)	28	4250	18,592	12,190,243	4.32	2.98	4.30	70.86	2.10	1.41	4.29	2.96
Chromaturia (PT: 10,008,796)	7	4271	3104	12,205,731	6.44	3.07	6.44	32.07	2.68	0.91	6.42	3.06
Hepatobiliary disorders (SOC: 10,019,805)												
Hepatotoxicity (PT: 10,019,851)	17	4261	4871	12,203,964	10.00	6.20	9.96	136.61	3.31	2.05	9.93	6.16
Hepatic cytolysis (PT: 10,049,199) *	14	4264	3404	12,205,431	11.77	6.96	11.74	136.99	3.55	2.02	11.69	6.91
Hepatitis (PT: 10,019,717)	8	4270	4131	12,204,704	5.54	2.76	5.53	29.61	2.46	0.91	5.52	2.76
Ear and labyrinth disorders (SOC: 10,013,993)												
Hypoacusis (PT: 10,048,865)	16	4262	11,153	12,197,682	4.11	2.51	4.09	37.39	2.03	1.09	4.09	2.50
Deafness (PT: 10,011,878)*	11	4267	5128	12,203,707	6.13	3.39	6.12	47.06	2.61	1.26	6.11	3.38
Vascular disorders (SOC: 10,047,065)												
Lymphoedema (PT: 10,025,282)	9	4269	1513	12,207,322	17.01	8.83	16.98	134.53	4.08	1.79	16.88	8.76
Skin and subcutaneous tissue disorders (SOC: 10,040,785)												
Photosensitivity reaction (PT: 10,034,972)	6	4272	2842	12,205,993	6.03	2.71	6.03	25.10	2.59	0.72	6.01	2.70
Nervous system disorders (SOC: 10,029,205)												
Vocal cord paralysis (PT: 10,047,674) *	5	4273	281	12,208,554	50.84	20.99	50.78	239.74	5.64	1.26	49.91	20.6
Central nervous system lesion (PT: 10,051,290)	4	4274	2031	12,206,804	5.62	2.11	5.62	15.17	2.49	0.25	5.61	2.10
Reproductive system and breast disorders (SOC: 10,038,604)												
Scrotal oedema (PT: 10,039,755)	4	4274	121	12,208,714	94.43	34.86	94.34	357.60	6.51	0.94	91.36	33.73
Infections and infestations (SOC: 10,021,881)												
Erysipelas (PT: 10,015,145)	3	4275	880	12,207,955	9.74	3.13	9.73	23.42	3.28	0.16	9.70	3.12

*New signals uncovered in the label and clinical trials.

**Figure 6:**
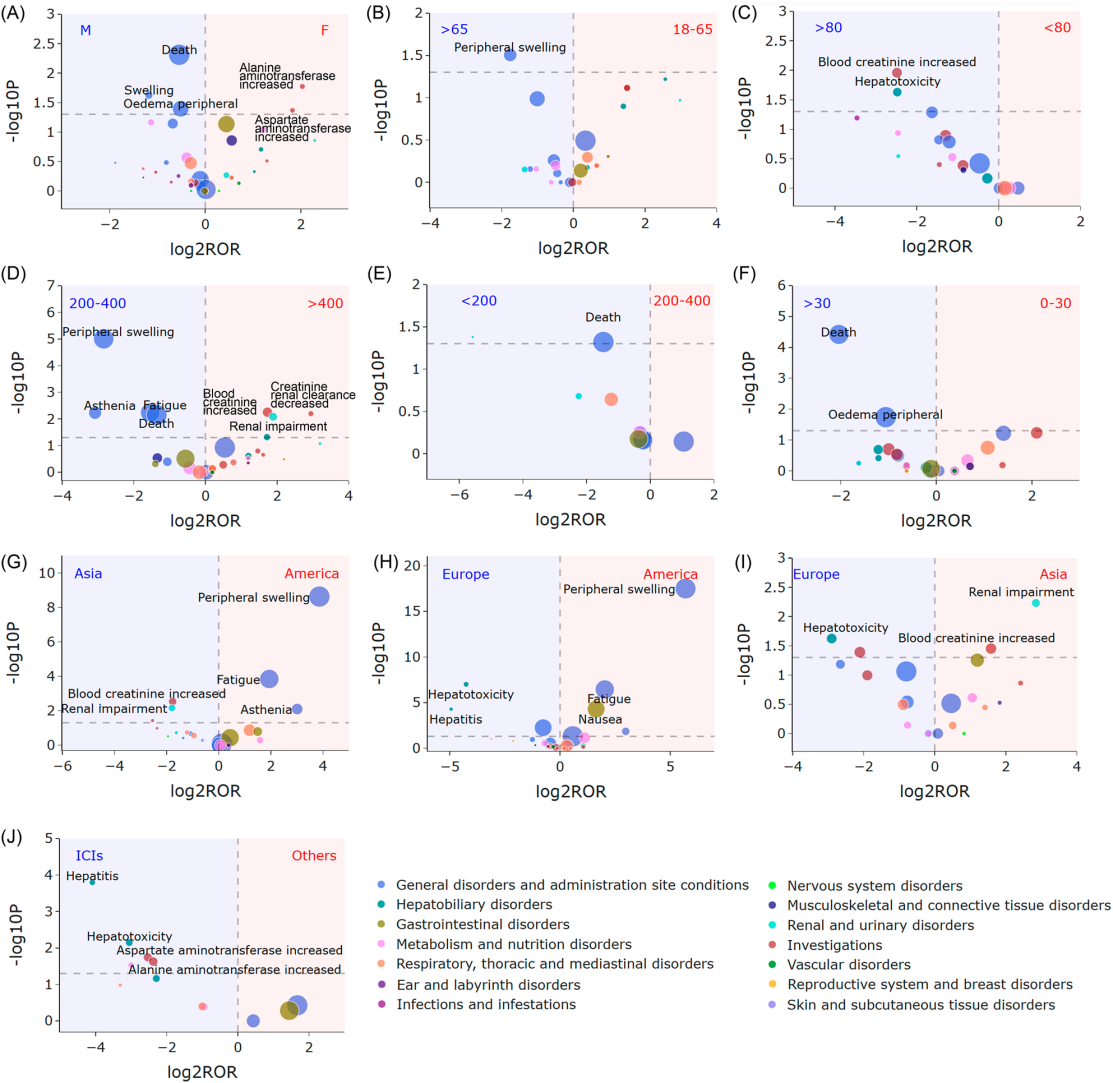
Volcano plots for differences detection of capmatinib signals. (**A**) Signal differences between females and males. (**B**) Signal differences between patients with age 18–65 years and > 65 years. (**C**) Signals differences between patients with weight < 80 kg and > 80 kg. (**D**) Signals differences between dose > 400 mg and 200–400 mg. (**E**) Signals differences between dose 200–400 mg and < 200 mg. (**F**) Signals differences between onset time 0–30 days and > 30 days. (**G**) Signals differences between occurred country in America and Asia. (**H**) Signals differences between occurred country in America and Europe. (**I**) Signals differences between occurred country in Asia and Europe. (**J**) Signals differences between cases combined with immune checkpoint inhibitors and those without. The x-axis is the logarithm of the ROR value (log2ROR) based on ROR algorithm, and the y-axis is the negative logarithm of the *P*-value calculated using Fisher’s exact test (− log10P). The colors of the individual points represent different SOCs. The sizes of the individual points represent the case numbers of each PT induced by capmatinib. In this volcano plot, signals within 49 significant disproportionality PTs are shown.

The volcano plots for differences detection of capmatinib signals are shown in Fig. 6. In these plots, larger y-values represent more strongly significant differences, while larger dot sizes represent higher signal frequencies at the PT level. Figure 6 reveals capmatinib signals exhibit distinct characteristics based on sex, age, weight, dose, onset time, continent, and concomitant drug. Notably, higher risk of alanine aminotransferase increased (PT: 10,001,551) and aspartate aminotransferase increased (PT: 10,003,481) were observed in females, especially when capmatinib was combined with immune checkpoint inhibitors. Compared with Europeans and Asians, Americans were more likely to experience peripheral swelling (PT: 10,048,959), especially in people > 65 years of age. Renal impairment (PT: 10,062,237) and blood creatinine increased (PT: 10,005,483) were more likely to occur in Asians and with single doses above 400 mg.

### Focus on death reports

**Figure 7:**
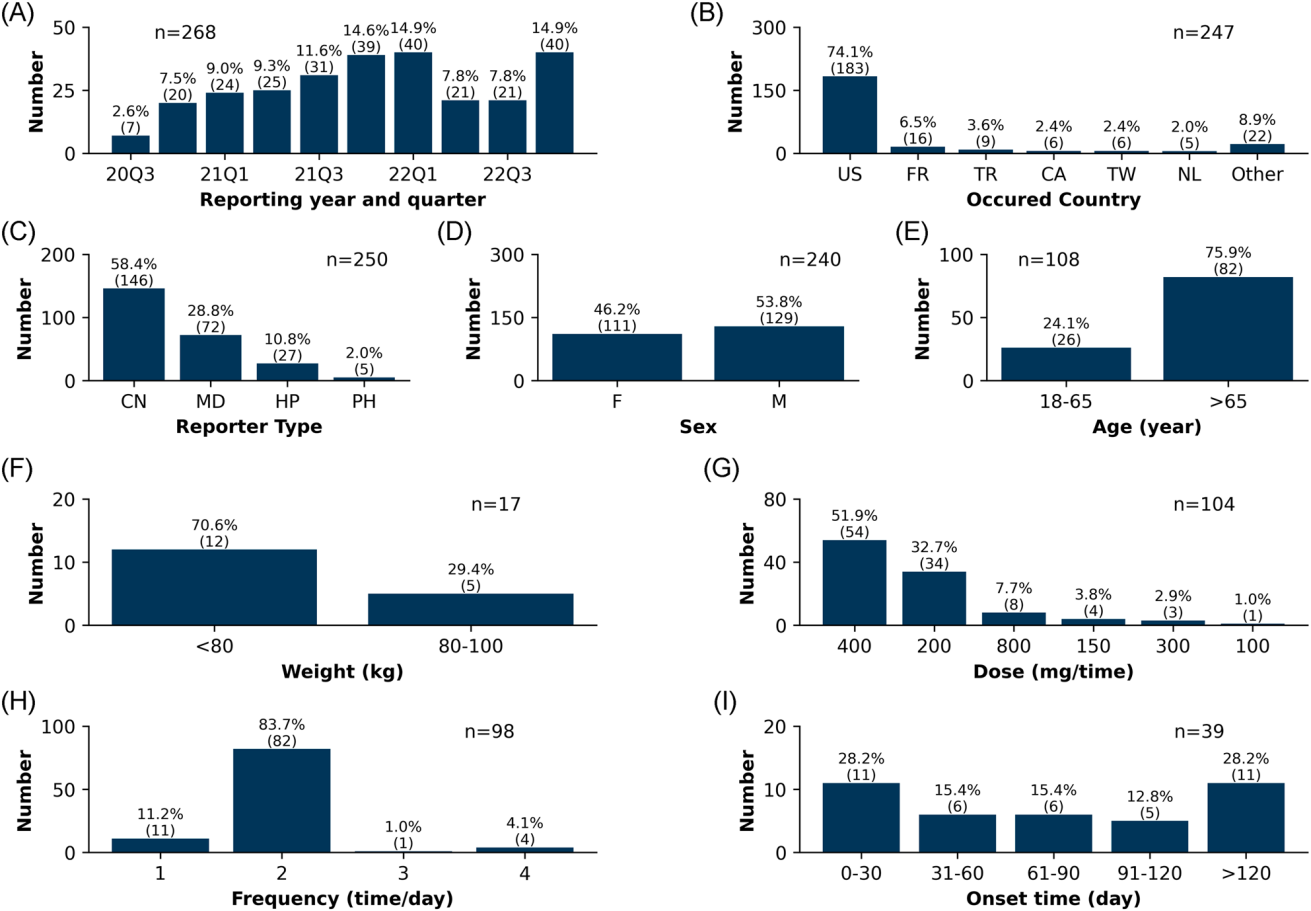
Clinical characteristics of capmatinib-associated death reports. (**A**) Reporting year and quarter. (**B**) Occurred country. (**C**) Reporter type. (**D**) Sex. (**E**) Age. (**F**) Weight. (**G**) Dose. (**H**) Frequency. (**I**) Onset time.

Death (PT: 10,011,906) was the signal with the highest number of reports. Special attention was paid on the 268 death reports. The clinical characteristics of the 268 capmatinib-associated death reports are shown in Fig. 7. By comparing the clinical characteristics of death reports with those of all adverse events, there were several findings. Firstly, in the death reports, no cases were found in Japan, which had a total of 30 cases of adverse events. In contrast, Turkey and Canada reported 9 and 6 cases of death, respectively, with a total of 18 and 11 adverse events, resulting in a death rate of 50% or higher. In terms of sex comparison, although the proportion of adverse events in females was higher than that in males, the proportion of death events in males was slightly higher than that in females. Furthermore, in reports with an onset time exceeding 60 days, the proportion of death events was higher than the overall proportion of adverse events. Although death may be caused by the underlying disease, these results suggest that special attention should be paid to serious adverse events occurred after 60 days of capmatinib administration in males in countries like Turkey and Canada.

## Conclusion

In this study, potential new adverse events were identified, which improve the understanding of safety profiles of capmatinib. Additionally, the adverse event signals of capmatinib exhibited distinct characteristics with different sexes, ages, weights, doses, onset times, continents, and concomitant drugs, which deserve special attention in clinical use.

## Discussion

Prior clinical researches have highlighted peripheral swelling as the most frequent adverse event linked to capmatinib. However, detailed stratified analysis between different populations remains unclear. This study found that compared with Europeans and Asians, Americans were more likely to experience peripheral swelling, especially in people > 65 years of age. Drug-induced peripheral swelling is typically non-inflammatory edema. Some reports showed MET pathway inhibitors and certain tyrosine kinase inhibitors (TKIs) like rilotumumab and onartuzumab also caused peripheral swelling^13,14^. The etiology is unknown but may be due to attenuated hepatocyte growth factor (HGF)/MET signaling in vascular endothelium, disrupting the balance and leading to leakage^15,16^. Therefore, early and vigilant monitoring such as weighing is recommended, which can reduce complications from managing late edema, especially in susceptible populations. Precautions like support stockings, bed elevation, reduced salt intake, and lymphedema massage should also be considered.

PTs of alanine aminotransferase increased and aspartate aminotransferase increased were also significant signals warranting discussion. The GEOMETRY mono1 trial of capmatinib revealed that 12% of patients experienced elevations in alanine aminotransferase (ALT), while 13% of patients experienced elevations in aspartate aminotransferase (AST)^10^. However, the patterns of subgroups were still ambiguous. This study found females were more likely to experience increased liver enzymes. The differences of capmatinib signals with respect to concomitant drug further revealed higher risk of ALT and AST increases when capmatinib was combined with immune checkpoint inhibitors. A recent study identified capmatinib-associated liver injury with portal fibrosis, with higher incidence after immunotherapy, supporting a potential interaction^17^. Given the widespread use of immune checkpoint inhibitors in clinical practice and the potential for liver enzyme elevation to cause severe consequences unnoticed by patients, thorough assessment of patients' immune checkpoint inhibitor history before capmatinib usage is critical. These findings suggest that clinicians should closely monitor liver function, especially in females and when capmatinib is combined with immune checkpoint inhibitors.

The differences of capmatinib signals with respect to dose further revealed that adverse events were intricately tied to the dose, with risks like increased creatinine, decreased renal clearance, and renal impairment higher at high doses. Typically, increased creatinine is often associated with renal damage, reflecting its severity. However, MET-TKIs can inhibit creatinine transporters, increasing levels without true impairment^18^. A case report described an 84-year-old on capmatinib with creatinine increasing from 1.6 to 2.4 mg/dL, but further evaluation found no renal impairment^18^. Therefore, oncologists should evaluate glomerular filtration rate accurately to distinguish this from true renal impairment. This prevents unnecessary premature discontinuation of capmatinib because of creatinine increasing.

This study further emphasized the importance of standardizing drug dosages. The recommended dosage for capmatinib is 400 mg orally twice daily. In cases of adverse reactions, dose reductions were recommended, with the initial reduction to 300 mg orally twice daily and the second reduction to 200 mg orally twice daily. However, this post-marketing surveillance study identified numerous instances of non-standard dosing practices in the United States, including once-daily dosing and single doses exceeding 400 mg or falling below 200 mg. It is important to note that this study, which relied on the FAERS database, did not enable an assessment of the dose and frequency received by patients treated with capmatinib but rather focused on patients who experienced adverse events. Without the total number of capmatinib patients, it is not possible to calculate the proportion of irrational use. The majority of the data in this study originated from the United States, as FAERS primarily includes adverse event reports from this country, with serious adverse event reports being available for other countries as well. Furthermore, capmatinib was used under temporary authorization in many countries and had not yet been approved for reimbursement. Consequently, limited data were available from these other countries, making it challenging to draw conclusions regarding deviations from recommended dosage specifications. Nonetheless, the study results underscore the existence of diverse nonstandard clinical dosages in the United States, which warrant clinical attention when using capmatinib.

Excitingly, this study identified several new signals, uncovered in the capmatinib label and unreported elsewhere. Vocal cord paralysis is a significant new signal which has diverse clinical presentations, often causing hoarseness, dysphagia, or choking from recurrent laryngeal or vagus nerve damage. It has been associated with drugs like vincristine, cisplatin, and nivolumab^19–21^. This study suggests that clinicians should focus on vocal cord paralysis when patients report hoarsening, misswallowing, or choking after receiving capmatinib. If necessary, laryngoscopy, laryngeal electromyography, imaging, voice acoustic analysis and other auxiliary diagnosis can be performed^22^.

In the current scenario, signal detection within the FAERS databases heavily relies on the application of disproportionality analysis methods, which are broadly categorized into two groups, including frequency count methods and Bayesian methods^23^. The former includes measures such as ROR, PRR, and the medicines and healthcare products regulatory agency (MHRA) algorithms, while the latter mainly involves BCPNN and MGPS algorithms^23–25^. However, each algorithm comes with its own limitations. A recent study suggested the utilization of correction algorithms to minimize the likelihood of false positive signal^26^. Nevertheless, as there are still unresolved issues with the current correction algorithms, such as the arbitrary choice of a threshold and a lack of explanation on how the chosen threshold reflects test correction, no common procedure was implemented to correct for multiple testing^26^. To mitigate potential biases, recent studies have adopted combinations of multiple algorithms in data analysis, such as combinations of ROR and BCPNN for detecting potential adverse events associated with ceftriaxone^27^, as well as combinations of ROR, PRR, BCPNN, and MGPS for quantifying signals associated with secukinumab^28^ and osimertinib^29^. Till now, there was no gold standard for handling the data from the FAERS databases. Ultimately, this study opted for two commonly-used frequency count methods (ROR and PRR) and two representative prominent Bayesian methods (BCPNN and MGPS) to explore potential adverse event signals of capmatinib.

This study has some limitations. Firstly, over 75% of the cases originated from the United States, and more than 50% of the reports were submitted by non-professionals (consumers), potentially introducing bias. Additionally, there were missing data for several variables in many reports, which could impact the results. Although this study employed different kinds of algorithms to reduce basis, the statistical tests cannot fully compensate for the limitations. Secondly, due to the limitations of spontaneous reporting systems for suspected adverse drug reactions, the duplicates are likely to remain with different CASEIDs. Currently, the literatures for managing the data from the FAERS database predominantly rely on the FDA-recommended deduplication method, which identifies deduplicating based on CASEID, PRIMARYID and FDA_DT. Consequently, this study also used this procedure to remove duplicates. However, duplicate data entries may still be left. Thirdly, all signal detection results merely indicate statistical correlations in which adverse events occurred and could not represent all cases in which capmatinib was used, necessitating further evaluation and research to ascertain the presence of a genuine causal relationship. Fourthly, several Traditional Chinese medicines have demonstrated effectiveness in treating NSCLC^30,31^. It is necessary to conduct further research to explore the effect of Traditional Chinese medicines combined with camatinib in alleviating adverse reactions. However, only a small number of cases in the FAERS database reported information on the concomitant drugs of Traditional Chinese medicine. While data mining technology cannot overcome the inherent limitations of the spontaneous reporting system or substitute expert reviews, it does play a significant role. Its outcomes can inspire medical professionals and patients alike, offering insights for subsequent research endeavors.

## Methods

### Data source and collection

The data for this retrospective drug vigilance study were extracted from the FAERS database, covering the third quarter of 2020 to the fourth quarter of 2022. Five types of datasets were used, including patient demographic and administrative information (DEMO), drug information (DRUG), the start and end dates of treatment with the reported drug (THER), adverse event encodings (REAC), and indication/diagnosis for use (INDI)^32–34^. Cases of capmatinib as the PS drug were identified using the generic name (prod_ai column as CAPMATINIB). All data were downloaded from the FDA website in ASCII format.

### Statistical analysis

Descriptive analysis methods were used to thoroughly characterize the clinical features of capmatinib-related adverse events after removing missing data, including reporting year and quarter, occurred country, reporter type, sex, age, weight, dose, frequency, and onset time^32–34^. Spearman correlation coefficient was used to explore correlations between typical clinical features, including sex, age, weight, dose, frequency, and onset time. For statistical analysis, the sex was encoded by assigning the males to 1 and the females to 0. T-test was further applied to assessing the differences in weight between females and males. As dose and frequency are critical factors for the rational use of capmatinib, the ANOVA test and sunburst plot were applied to analyzing the correlation of dose and frequency, as well as their distributions across countries. The concomitant drugs of the capmatinib-associated adverse event reports were then analyzed^32^. In the same report, if capmatinib was identified as the PS drug, the other drugs labeled as ‘secondary suspect’, ‘concomitant’, or ‘interacting’ were considered concomitant drugs^32^.

The adverse events were coded using PTs, which were mapped to the corresponding primary SOC level based on the standardized Medical Dictionary for Regulatory Activities (MedDRA) version 25.1. The adverse event signals of capmatinib were investigated by profiling the frequency and intensity at the SOC and PT levels^32–34^. Four disproportionality analysis methods were used, including ROR^35,36^, PRR^29,33^, BCPNN^23,29,33^, and MGPS^23,29,33^. The fourfold table of disproportionality analysis, equations and criteria of the four algorithms for capmatinib signal detection are shown in Supplementary Table S2–S3. In this study, a PT was considered as an effective adverse event signal if it met the criteria of four algorithms concurrently^32–34^. Since all PTs were collected from FAERS, signals related to neoplasms benign, malignant and unspecified (incl cysts and polyps) (SOC: 10,029,104), product issues (SOC: 10,077,536), and injury, poisoning and procedural complications (SOC: 10,022,117), as well as the signal of disease progression (PT: 10,061,818), were excluded. Additionally, capmatinib-associated adverse event signals were investigated with respect to sex, age, weight, dose, onset time, continent, and concomitant drug using the ROR algorithm and Fisher’s exact test^32^. The fourfold table, criteria of ROR and Fisher's exact test for difference detection of capmatinib signal are shown in Supplementary Tables S4–S5. All data processing and statistical analyses were performed using Jupyter Notebook 6.4.12, providing a Python 3 (ipykernel) computational environment.

### Supplementary Information

Supplementary Tables.

## Data Availability

The datasets analyzed during the current study are available from the corresponding author on reasonable request.
