# Supplementary material for: A real-world pharmacovigilance study of FDA adverse event reporting system events for Capmatinib
Source: Sci Rep. 2024 May 18;14:11388. doi: 10.1038/s41598-024-62356-w (PMC11102445; doi:10.1038/s41598-024-62356-w)
Supplement: Supplementary file 1 — Supplementary Tables. [file 41598_2024_62356_MOESM1_ESM.docx]

Supplementary Material

### A Real-World Pharmacovigilance Study of FDA Adverse Event Reporting System Events for Capmatinib

**Supplementary Table S1.** Case number and signal strength of capmatinib-unrelated adverse events at the PT level.

| **PT** | **a** | **b** | **c** | **d** | **ROR** | | **PRR** | | **BCPNN** | | **MGPS** | |
| --- | --- | --- | --- | --- | --- | --- | --- | --- | --- | --- | --- | --- |
|  |  |  |  |  | **ROR** | **Lower limit of 95% CI** | **PRR** | **χ2** | **IC** | **IC025** | **EBGM** | **EBGM05** |
| **Neoplasms benign, malignant and unspecified (incl cysts and polyps) (SOC: 10029104)** | | | | | | | | | | | | |
| Malignant neoplasm progression (PT: 10051398) | 97 | 4181 | 20855 | 12187980 | 13.56 | 11.08 | 13.27 | 1097.65 | 3.72 | 3.26 | 13.22 | 10.80 |
| Non-small cell lung cancer (PT: 10061873) | 56 | 4222 | 1014 | 12207821 | 159.69 | 121.81 | 157.61 | 8259.15 | 7.22 | 4.98 | 149.41 | 113.98 |
| Lung neoplasm malignant (PT: 10058467) | 21 | 4257 | 19182 | 12189653 | 3.13 | 2.04 | 3.12 | 30.35 | 1.64 | 0.89 | 3.12 | 2.03 |
| Metastases to central nervous system (PT: 10059282) | 15 | 4263 | 2413 | 12206422 | 17.80 | 10.70 | 17.74 | 235.54 | 4.14 | 2.39 | 17.64 | 10.61 |
| Non-small cell lung cancer metastatic (PT: 10059515) | 13 | 4265 | 102 | 12208733 | 364.83 | 204.68 | 363.73 | 4170.95 | 8.33 | 2.93 | 322.72 | 181.06 |
| Metastases to bone (PT: 10027452) | 9 | 4269 | 3347 | 12205488 | 7.69 | 3.99 | 7.67 | 52.11 | 2.94 | 1.29 | 7.66 | 3.98 |
| Lung adenocarcinoma (PT: 10025031) | 8 | 4270 | 557 | 12208278 | 41.06 | 20.42 | 40.99 | 307.70 | 5.34 | 1.94 | 40.42 | 20.10 |
| Neoplasm (PT: 10028980) | 5 | 4273 | 2491 | 12206344 | 5.73 | 2.38 | 5.73 | 19.48 | 2.52 | 0.50 | 5.72 | 2.38 |
| Brain neoplasm (PT: 10061019) | 4 | 4274 | 1964 | 12206871 | 5.82 | 2.18 | 5.81 | 15.91 | 2.54 | 0.27 | 5.80 | 2.17 |
| Metastasis (PT: 10062194) | 3 | 4275 | 1327 | 12207508 | 6.46 | 2.08 | 6.45 | 13.79 | 2.69 | 0.00 | 6.44 | 2.07 |
| Lung cancer metastatic (PT: 10050017) | 3 | 4275 | 455 | 12208380 | 18.83 | 6.05 | 18.82 | 50.28 | 4.22 | 0.34 | 18.7 | 6.01 |
| Metastases to adrenals (PT: 10027451) | 3 | 4275 | 175 | 12208660 | 48.96 | 15.63 | 48.92 | 138.46 | 5.59 | 0.45 | 48.12 | 15.36 |
| **Product issues (SOC: 10077536)** | | | | | | | | | | | | |
| Product availability issue (PT: 10077800) | 10 | 4268 | 6779 | 12202056 | 4.22 | 2.27 | 4.21 | 24.45 | 2.07 | 0.83 | 4.21 | 2.26 |
| Product supply issue (PT: 10077801) | 8 | 4270 | 2621 | 12206214 | 8.73 | 4.36 | 8.71 | 54.45 | 3.12 | 1.26 | 8.69 | 4.34 |
| **Injury, poisoning and procedural complications (SOC: 10022117)** | | | | | | | | | | | | |
| Product use complaint (PT: 10079400) | 13 | 4265 | 4148 | 12204687 | 8.97 | 5.20 | 8.94 | 91.47 | 3.16 | 1.74 | 8.92 | 5.17 |
| **Others** | | | | | | | | | | | | |
| Disease progression (PT: 10061818) | 38 | 4240 | 23694 | 12185141 | 4.61 | 3.35 | 4.58 | 106.26 | 2.19 | 1.60 | 4.57 | 3.32 |

**Supplementary Table S2**. Fourfold table for capmatinib signal detection.

|  | Reports with target adverse event | Reports with other adverse events |
| --- | --- | --- |
| Reports with capmatinib | a | b |
| Reports with other drugs | c | d |

a, number of reports that include target adverse event associated with capmatinib; b, number of reports that include other adverse events associated with capmatinib; c, number of reports that include target adverse event associated with other drugs; d, number of reports that include other adverse events associated with other drugs.

**Supplementary Table S3.** Equations and criteria of four algorithms for capmatinib signal detection.

| Algorithms | Equation | Criteria |
| --- | --- | --- |
| ROR | ROR=(ad)/(bc) | Lower limit of 95% CI>1, N≥2 |
|  | Lower limit of 95% CI=e^ln(ROR)-1.96(1/a+1/b+1/c+1/d)^0.5^  Upper limit of 95% CI=e^ln(ROR)+1.96(1/a+1/b+1/c+1/d)^0.5^ |  |
| PRR | PRR=a(c+d)/c/(a+b) | PRR≥2, χ2≥4, N≥3 |
|  | χ2=[(ad-bc)^2](a+b+c+d)/[(a+b)(c+d)(a+c)(b+d)] |  |
| BCPNN | IC=log_2_a(a+b+c+d)/(a+c)/(a+b) | IC025>0 |
|  | IC025=E(IC)-2V(IC)^0.5 |  |
| MGPS | EBGM=a(a+b+c+d)/(a+c)/(a+b) | EBGM05>2 |
|  | EBGM05=e^ln(EBGM)-1.96(1/a+1/b+1/c+1/d)^0.5^ |  |

N, number of reports; 95% CI, 95% confidence interval; IC, information component; IC025, lower limit of 95% confidence interval of IC; E(IC), IC expectation; V(IC), variance of IC; EBGM, empirical Bayesian geometric mean; EBGM05, lower limit of 95% confidence interval of EBGM.

**Supplementary Table S4**. Fourfold table for difference detection of capmatinib signals.

|  | Reports with target adverse event | Reports with other adverse events |
| --- | --- | --- |
| Factor 1 | a | b |
| Factor 2 | c | d |

a, number of reports that include the target adverse event, with capmatinib identified as the primary suspected drug, conforming to factor 1; b, number of reports that include other adverse events, with capmatinib identified as the primary suspected drug, conforming to factor 1; c, number of reports that include the target adverse event, with capmatinib identified as the primary suspected drug, conforming to factor 2; d, number of reports that include other adverse events, with capmatinib identified as the primary suspected drug, conforming to factor 2.

**Supplementary Table S5**. Criteria of ROR and Fisher’s exact test for difference detection of capmatinib signals.

|  | ROR | Fisher’s exact test |
| --- | --- | --- |
| Signals that factor 1 more likely to develop | ROR>1, Lower limit of 95% CI>1,  N≥2 | P<0.05 |
| Signals that factor 2 more likely to develop | ROR<1, Upper limit of 95% CI<1,  N≥2 | P<0.05 |
